# “Rule‐Following I: The Basic Issues”

**DOI:** 10.1111/phc3.12900

**Published:** 2023-01-26

**Authors:** Indrek Reiland

**Affiliations:** ^1^ Department of Philosophy University of Vienna Vienna Austria

## Abstract

‘Rule‐following’ is a name for a cluster of phenomena where we seem both guided and “normatively” constrained by something *general* in performing particular actions. Understanding the phenomenon is important because of its connection to meaning, representation, and content. This article gives an overview of the philosophical discussion of rule‐following. Part I of this two‐part contribution is devoted to the basic issues from Wittgenstein to Kripkenstein's skeptical paradox. Part II will be about recent answers to the skeptical paradox and Boghossian's and Wright's new puzzles.

## 1 INTRODUCTION

‘Rule‐following’ is a name for a cluster of phenomena where we seem both guided and “normatively” constrained by something *general* in performing particular actions. Take the action of continuing a series like 2, 4, 6, 8… by following the “rule” +*2* and writing down 1002. It seems that the rule *+2* both guides and constrains our action: it is what leads us to write down 1002 and makes this *correct*, while making any other way of continuing the series incorrect.

Understanding the phenomenon is important because of its connection to meaning, representation, and content. It's easiest to see this by highlighting the common assumption that representation involves *attribution* or *predication* of general properties like redness (Burge, 2010; Hanks, 2015; Soames, 2010). Predication is analogous to sorting. Take the activity of sorting marbles into two piles based on whether they're red or not. It seems that our *use* of the property of redness as our principle of sorting both *guides* and *constrains* our actions: it is what leads us to put a particular red marble in the red pile and makes this correct, while making putting a green one in the red pile incorrect.

Rule‐following raises several questions of which the following two are the central focus of contemporary discussion:

Q1) What does our *grasp* of a particular rule (rather than another one) consist in?
Q2) How do we move from our grasp of a rule to its *application* to a particular case?

Plausible answers to these questions have been thought to have a range of important consequences: that rule‐following is only possible in the context of a practice, perhaps a necessarily social one (Wittgenstein, 1953); that rule‐following doesn't consist in a fact, even though rule‐following talk is fine (Kripke, 1982); that rule‐following is a primitive phenomenon (Boghossian, 1989; McDowell, 1984; Stroud, 2000).

This article gives an overview of the philosophical discussion of rule‐following with emphasis on Kripke's skeptical paradox and recent work on possible solutions.

Part I of this two‐part contribution is devoted to the basic issues from Wittgenstein to Kripke, the latter of whom focused on Q1. Part II will be about recent answers to the skeptical paradox and Boghossian's and Wright's new puzzles which focus on Q2.

## 2 PART I: THE BASIC ISSUES FROM WITTGENSTEIN TO KRIPKE

### 2.1 Rules, ‘Normativity’, and Following

What are rules in this context and what is it to follow them? A rule in this context is anything general that can be *followed* such that:

a) our actions can *accord* or *discord* with it, in some sense (*conformity*);
b) our having, grasp, or use of it can play a role in *generating* and *explaining* our action (*guidance*).

Functions like +2 and properties like redness both count as rules in this sense when *used* as principles for continuing a series or sorting. One *conforms* with a rule when one acts in accordance with it. This is possible even without any sensitivity to its demands. One is *guided* by a rule when one *tries* to act in accordance with it or one's action is somehow otherwise a product of sensitivity to its demands. This is possible while failing to conform. One *follows* the rule when one is both guided by it and conforms with it.

There are two important issues to get clear on. The first concerns *accord/discord* and *conformity*. These are frequently claimed to be ‘normative’ properties or statuses. However, we must be careful not to build too much into this claim. ‘Normativity’ is here to be understood very cheaply, just in the sense that some ways of acting count as *correct* and others as *incorrect* relative to the rule (contrast Hattiangadi, 2007, Ch. 3, Ch. 7). Correctness is plausibly a type of normative property in some sense, but it's an open question how it relates to normative properties in a richer sense used in metaethics, for example to deontic and evaluative properties.^1^ Any richer characterization of the normativity involved, for example, in terms of deontic notions, will be contentious and not be plausible across the board. For example, even if it could be argued to apply in the case of linguistic meaning or explicit, intentional rule‐following, it is not clear it applies in the case of representation and content. This also means that the notion of a rule in play in the discussion of rule‐following is a lot broader than the more familiar and intuitive notion of rule in play in philosophy of law where the accord/discord is standardly thought to be deontic (involving *must‐s*, *can't‐s*, and *may‐s*) (Reiland, 2020). In other words, many things that count as rules in the context of rule‐following, like functions and properties used as principles for doing things, do not count as rules in the narrower sense of philosophy of law.

The second thing we need to get clear on are *guidance* and *following*. Are these to be understood in an *intentional* manner? This is an important choice point. On the one hand, in most ordinary examples of rule‐following that people give we do follow rules intentionally, by *trying* to act in accordance with them (Boghossian, 2012; Pettit, 1990). On the other hand, if intentional rule‐following itself depends on contentful states, as is plausible, then representation and content can't be understood in terms of it. Thus, if rule‐following is thought to be necessarily intentional then the topic loses its connection to representation and content. This is why philosophers like Sellars and his followers think of rule‐following in a non‐intentional manner, in terms of some sort of non‐agentive sensitivity to the rule's demands (Sellars, 1954; Stovall, 2021). One should therefore always try to get clear on whether the sort of rule‐following discussed is thought to be necessarily intentional or possibly non‐intentional. This should also be kept firmly in mind in thinking about Kripke's discussion and its scope and we will return to this point in Part II.

### 2.2 Wittgenstein

Wittgenstein's thoughts about rule‐following are mainly contained in his *Philosophical Investigations* (1953) and *Remarks on the Foundations of Mathematics* (1956). Here we have space only for a brief overview of his discussion of rule‐following in ##185‐202 of the former, mainly to serve as a foil to Kripke's discussion. Since definite conclusions are impossible to arrive at, we'll stay relatively close to the text and look at some main strands of interpretation.

In #185 Wittgenstein gives the example of a pupil who has been taught to continue the series 2, 4, 6, 8… by *+2* and continues until 1000, after which she carries on by writing down 1004, 1008, 1012. Furthermore, she insists that she is going on the same way as before. One simple moral of this story is that any actual pattern of behavior *conforms* with an infinite number of rules, in this case with both *+2* and with *+2 if n > 1000 and + 4 if n < 1000*. Thus, regular, patterned behavior is by itself insufficient to determine which rule the teacher tries to impart or which one the pupil is following, if any. This is what Brandom calls the *gerrymandering* argument (Brandom, 1994, pp. 28‐29). Another way of putting it is that any pattern of behavior can be *multiply interpreted*.

One might therefore think that what the teacher should do is produce a series and give some further information that specifies what rule they have in mind. What sort of information? If the teacher adds just more examples then this is of no help. However, and this is the second moral of the story, an added *interpretation* in the sense of explicit instructions in language won't help either since those can be multiply interpreted as well. This is what is standardly known as the *regress* argument (Brandom, 1994, pp. 20‐21, McDowell, 1992, pp. 265‐266).

On the flipside, we shouldn't think that the student's grasp of one rule rather than another consists in an added *interpretation* either. Again, this is because any such thing can be multiply interpreted (for discussion, see Child, 2011, pp. 134‐136, McGinn, 1984, pp. 13‐19). Wittgenstein's conclusion is that thinking of grasping a rule in terms of an added interpretation is a mistake:

198. “But how can a rule teach me what I have to do at *this* point? After all, whatever I do can, on some interpretation, be made compatible with the rule.” – No, that’s not what one should say. Rather, this: every interpretation hangs in the air together with what it interprets, and cannot give it any support. Interpretations by themselves do not determine meaning.^2^

Instead, there must be a way of grasping a rule that is not a matter of adding an interpretation to it and that is achieved by training:

198. … “So is whatever I do compatible with the rule?” – Let me ask this: what has the expression of a rule – say a signpost – got to do with my actions? What sort of connection obtains here? – Well, this one, for example: I have been trained to react in a particular way to this sign, and now I do so react to it.

But with this you have pointed out only a causal connection; only explained how it has come about that we now go by the signpost; not what this following‐the‐sign really consists in. Not so; I have further indicated that a person goes by a signpost only in so far as there is an established usage, a custom.

199. Is what we call “following a rule” something that it would be possible for only *one* person, only *once* in a lifetime, to do? – And this is, of course, a gloss on the *grammar* of the expression “to follow a rule”.

It is not possible that there should have been only one occasion on which only one person followed a rule. It is not possible that there should have been only one occasion on which a report was made, an order given or understood, and so on. – To follow a rule, to make a report, to give an order, to play a game of chess, are *customs* (usages, institutions).

Here Wittgenstein first emphasizes that any pattern of behavior or *expression* of a rule such as a signpost or an explicit instruction is connected to our actions through training. Furthermore, he insists, this isn't to just give a causal story, but also to point out that the *grammar* of our talk of rule‐following, our concept of rule‐following, is such that talk of it only makes sense in the context of an enduring practice.^3^

The whole discussion is summed up in the following paragraph:

201. This was our paradox: no course of action could be determined by a rule, because every course of action can be brought into accord with the rule. The answer was: if every course of action can be brought into accord with the rule, then it can also be brought into conflict with it. And so there would be neither accord nor conflict here.

That there is a misunderstanding here is shown by the mere fact that in this chain of reasoning we place one interpretation behind another, as if each one contented us at least for a moment, until we thought of yet another lying behind it. For what we thereby show is that there is a way of grasping a rule which is *not* an interpretation, but which, from case to case of application, is exhibited in what we call “following the rule” and “going against it”.

Here Wittgenstein says that the seeming paradox generated by the fact that neither regular, patterned behaviour nor added interpretations are sufficient to determine which rule one is following, is based on a misunderstanding. The real lesson here is that there must be a way of grasping a rule that is not a matter of adding an interpretation and that is achieved by training that institutes one into an enduring practice (for discussion see Child, 2011, pp. 146‐142, Fogelin, 2009: Ch. 1; for a distinctive gloss on what this entails see Ginsborg, 2020). This is generally thought to be a counterpoint and corrective to Kripke's reading, who presented Wittgenstein as thinking that the paradox is real and in need of solving (McDowell, 1992, p. 255; McGinn, 1984, pp. 67‐69).

Can an individual have an established practice of their own or must the practice be social? Wittgenstein at least seems to think the latter:

202. That’s why ‘following a rule’ is a practice. And to *think* one is following a rule is not to follow a rule. And that’s why it’s not possible to follow a rule ‘privately’; otherwise, thinking one was following a rule would be the same thing as following it.

The essential point is that rule‐following is enmeshed with ‘normativity’ and the difference between correctness/incorrectness. This means that there must be a gap between thinking that one is following a rule and actually following it. Wittgenstein at least seems to claim that this means that private rule‐following is impossible, but this is open to interpretation and subject to disagreement (for discussion see Child, 2011, pp. 142‐146, McGinn, 1984, pp. 77‐92).

There are several further themes in Wittgenstein's ensuing discussion. First, that we don't ourselves have more than we can impart in teaching (##208‐210). Second, and relatedly, that in our own case we run out of reasons in explaining and justifying why we do what we do, and therefore do so blindly, without reasons (##211‐219). Finally, that rule‐following only makes sense against the context of a shared form of life (##206, 241‐242). However, we will leave Wittgenstein now and move to Kripke's influential interpretation which is the centerpiece of all current discussion.

### 2.3 Kripkenstein

Kripke's *Wittgenstein on Rules and Private Language* (1982) brought Wittgenstein's concerns forcefully into mainstream focus by offering a particular reading of them that leads to a skeptical paradox. Because Kripke presented himself as merely expounding a line of argument, for ease of discussion the thoughts are usually attributed to a fictional protagonist called Kripke's Wittgenstein or Kripkenstein.

Kripkenstein's main question, generalized, is:

1) What does our *grasp* of a rule (rather than another one) consist in?

However, Kripkenstein doesn't present things directly in terms of grasping a rule but in terms of a speaker's using a word to denote something or meaning something by a word. For example, he talks about using ‘plus’ or ‘+’ to denote the addition function or meaning the property of *being a table* by ‘table’ (Kripke, 1982, pp. 7, 19). This raises a host of questions. By using constructions like ‘I mean *addition* by ‘plus’’ is Kripkenstein talking about linguistic meaning in a speaker's idiolect or something else? Do the issues look different when one thinks that public language linguistic meaning is prior to idiolectal meaning? Does the argument extend from language to thought, from meaning to representation and content? How does all of this generalize to rule‐following more broadly?

I think the most natural reading is that Kripkenstein is indeed talking about linguistic meaning in a speaker's idiolect. Given this assumption the *skeptical challenge* is the following. I'm confident that ‘plus’ has a meaning in my idiolect which associates it with applying the addition function. This is what the shorthand “I mean *addition* by ‘plus’” stands for. Now, suppose I've never used the expression ‘68 plus 57 = *x*’ before. Kripkenstein then posits a skeptic who asks what about my past behavior or in my mind right now makes it the case that ‘plus’ is indeed associated with applying the addition function and thus, to use it correctly, in both the linguistic and factual sense, I must replace *x* with 125?^4^ After all, given that I've never used the expression before, everything I've done in the past seems compatible with ‘plus’ having a meaning in my idiolect which associates it with applying the quaddition function instead, which is defined as follows:

$$\begin{array}{ccc} x\;quus\;y & = & x\;plus\;y,\;if\;x,\;y\;<\;57\\ & = & 5\;otherwise \end{array}$$

And, of course, if it is instead associated with applying the quaddition function then to use the word correctly, in both senses, I must answer not 125 but 5. Thus, the skeptical challenge is to explain what it is for it to be the case that ‘plus’ in my idiolect has a meaning that associates it with applying the addition function rather than the quaddition function.

It is important to understand that there's nothing special about ‘plus’ that generates this challenge and in many ways the mathematical example makes things messier than they need to be. I'm also confident that in my idiolect ‘is a table’ has a meaning which associates it with predicating the property of *being a table* of things. Now, suppose I've never used the sentence ‘This is a table’ while pointing to a table at the base of the Eiffel tower. We can again ask what in my past behavior or in my mind right now makes it the case that ‘is a table’ is indeed associated with predicating the property of *being a table*. After all, given that I've never used the sentence at the base of the Eiffel tower, everything I've done in the past seems compatible with ‘is a table’ having a meaning in my idiolect which associates it instead with predicating the property of being a *tabair* where a tabair is “anything that is a table not found at the base of the Eiffel tower or a chair found there” (Kripke, 1982, p. 19). Thus, the skeptical challenge could also be put by saying that it is to explain what it is for it to be the case that ‘is a table’ in my idiolect has a meaning that associates it with predicating the property of *being a table* rather than the property of *being a tabair*. In general terms, it is to explain *what it is for an expression to have a particular meaning in a speaker's idiolect (rather than another)* (Kripke, 1982, p. 11).

Kripkenstein stresses that a proper answer to this question is subject to an important constraint. The answer must show how the expression's having a particular meaning in one's idiolect both *guides* one in and, in some sense, linguistically *justifies* one's word use, makes it rational from your first‐person point of view. As Kripkenstein puts it, the answer must show how, “I'm justified in giving the answer ‘125’ to ‘68 + 57’”, that this “is the answer I ought to give” (Kripke, 1982, p. 11). Usually, when we're faced with ‘68 + 57 = *x*’, we feel like it's the meaning of ‘plus’ together with the calculation we perform that *guides* us in answering 125. The answer to the skeptic must make clear how the fact that my meaning addition by ‘plus’ consists in can guide me and make it linguistically justified by my own lights, from the inside (for recent discussion see Ginsborg, 2022, Sultanescu, 2022).^5^ We can call this the *Guidance* constraint, and it should be immediately clear that it is much more plausible for language use and intentional rule‐following than representation and non‐intentional rule‐following.

We don't have space here to go deeper into whether the challenge looks different if one thinks that public language linguistic meaning is prior to idiolectal meaning (for a brief discussion see Boghossian, 1989, pp. 509‐510, 2012, pp. 37‐38; Hattiangadi, 2007, pp. 14‐15). However, it is not hard to see how a version of the argument could extend from meaning to representation and content. Let's assume that to represent *o* as being a table is to predicate the property of *being a table* of it (Burge, 2010; Hanks, 2015; Soames, 2010). Then we can ask what does your predicating the property of *being a table* of *o* rather than the property of *being a tabair* consist in. In other words, what does your mind's use of a particular property as a principle of predication consist in (for discussion see Hanks, 2017)? And since a property used as a principle of predication is just one example of a rule, in the present sense, it's easy to see how this generalizes to all rule‐following. What isn't clear, as we will see in Part II, is whether anything like the *Guidance* constraint generalizes to representation and non‐intentional rule‐following.

Having presented the skeptical challenge, Kripkenstein goes through several possible answers and disposes of them one by one. Note that here we're just looking at his discussion, it is in Part II where we'll look at contemporary elaborations of the answers in more depth.

So, what is it for ‘plus’ to have a meaning in my idiolect that associates it with applying the addition function? In shorthand, what is it for me to mean *addition* by ‘plus’?

a) *Explicit instructions*: the first answer is that I didn't just extrapolate from a set of finite examples of applying ‘+’ to pairs of numbers, I gave myself explicit instructions. For example, that to get ‘x + y’ you count x amount of marbles and y amount of marbles, then put them together and count the total, which is the sum. The response is, predictably, that such explicit instructions can themselves be multiply interpreted and so the question now just becomes what makes it the case that ‘count’ is for talking about *counting* rather than *quounting*, where to quont is… (Kripke, 1982, pp. 15‐17)
b) *Dispositions*: the second answer is that it is constituted by my dispositions to use the word in a particular way. For example, to use it to give the *sum* and not the *quum.* The response is threefold. First, the totality of our dispositions to use words are finite. There are some numbers that are so large that our minds can't grasp them and as such we're not disposed to give their sum when queried. However, our word ‘plus’ nevertheless applies to them and yields the sum. Thus, the dispositionalist answer doesn't respect the infinitary character of meaning.

Second, our dispositions to use words include dispositions to make what intuitively count as mistakes. For example, we might be disposed to forget to carry when asked to add certain numbers and therefore disposed to give what intuitively counts as the wrong answer. But if our dispositions are treated as constitutive of meaning then there is no basis for taking this to be the wrong answer rather than taking us to be applying a different function. Thus, the dispositionalist answer can't make sense of the idea that we can make mistakes.

Finally, and most importantly, the answer in terms of dispositions is beside the point since it can't make sense of the dual ideas of a normative constraint and guidance. If ‘plus’ is associated with applying the addition function then what I must do if I'm to use the word correctly, in accordance with its meaning, is to use it to apply the addition function rather than the quaddition function. And it is my meaning addition by ‘plus’ that also guides me in my use and makes it justified. But our dispositions merely determine how we will *in fact* use the word (Kripke, 1982, pp. 24‐25).

c) *Simplicity*: the third answer tries to appeal to the idea that addition is simpler than quaddition. The response is that this mistakes a metaphysical problem for an epistemological one. The question is what it is for ‘plus’ to have a particular meaning in my idiolect. We're in search for a constitutive answer. Simplicity considerations *by themselves* cannot provide such an answer, but only help us decide between several competing epistemological hypotheses about what the actual meaning in fact is (Kripke, 1982, p. 38).
d) *Experience*: the fourth answer is that my meaning addition by ‘plus’ is a special experience with its own irreducible quale. The response is twofold. First, as in the case of dispositionalism, this answer is again beside the point since it can't make sense of the dual ideas of normative constraint and guidance. In other words, it can't make sense of the fact that some uses are correct and others incorrect and that we're guided by meaning. The situation is similar than in the case of the classical empiricist idea that meaning the property of being a table by ‘table’ consists in the fact that when one uses the word a mental image of a table comes to one's mind. The problem is that such images are multiply interpretable and don't make any use of the word correct or incorrect, nor can they guide us (for discussion, see Child, 2011, pp. 108‐111; McGinn, 1984, pp. 13‐19). And the same applies to the answer in terms of a special experience with its irreducible quale. As Kripke puts it, such a state “would not tell me what to do in new cases” (Kripke, 1982, p. 43). Second, if we look close enough, we see that there is no such special experience characteristic of meaning *addition* rather than *quaddition* (Kripke, 1982, pp. 44‐46).
e) *Primitivism*: the fifth answer is that my meaning addition by ‘plus’ is a primitive non‐qualitative state that can't be further explained. The response is short: this move is desperate and leaves the supposed state mysterious. It would have to be a finite state that reaches out to infinity and that is hard to wrap our minds around (Kripke, 1982, pp. 51‐52). Nevertheless, Kripke himself expresses some sympathy to this view: “Even now I have a strong inclination to think this somehow must be right.” (Kripke, 1982, p. 52).
f) *Platonism*: the final answer seeks help from Platonism about rules, functions, and properties coupled with Frege's view of how meaning determines reference. For example, the *addition* function is something that by its nature takes 57 and 68 as inputs and yields 125 as an output. And on Frege's view, ‘plus’ picks out *addition* because it expresses a sense PLUS which by its nature determines it as its reference. On this view there is no problem as to how PLUS refers to *addition* nor how the function as applied to 57 and 68 yields 125 as output. However, the predictable response is that this doesn't really address the original question since it doesn't tell us how ‘plus’ has a meaning in my idiolect that expresses the sense PLUS rather than QUUS. As Kripke puts it: “For Wittgenstein, Platonism is largely an unhelpful evasion of the problem…Platonic objects might be self‐interpreting… but ultimately there must be some mental entity involved which raises the sceptical problem.” (Kripke, 1982, p. 54).

After having disposed of all the answers, Kripkenstein arrives at the *skeptical conclusion* that meaning something by something or rule‐following doesn't consist in anything. He then proceeds to give a *skeptical solution* which, on its most standard interpretation, rejects the cognitivist or factualist presupposition that talk of someone's meaning something particular by a word is fact‐stating. Instead, we should think of the meaning of sentences in general in terms of the conditions in which they can be correctly used (e. g. their use‐conditions) and the role they play in our lives (Kripke, 1982, pp. 73‐74). The use‐conditions of first‐personal meaning ascriptions like ‘I mean *addition* by ‘plus’’ are that one has to be confident that one knows how to use it. However, the use‐conditions of third‐personal meaning ascriptions like ‘Jones means *addition* by ‘plus’’ are that Jones's use has to agree with yours (Kripke, 1982, pp. 91‐92). And the role of such sentences is to mark out the people who can be relied on to use the expressions in the same way we do.

How to exactly understand the skeptical solution is subject to controversy, but further coverage of it is beyond the scope of this overview (for discussion see Hattiangadi, 2007: Ch. 4, Miller, 2020, Wilson, 2002). In Part II we will instead discuss recent work on the above solutions, including Ginsborg's novel view, and Boghossian's and Wright's new puzzles.

## ENDNOTES

^1^
It's a platitude that a belief or judgment that p is semantically, or, better, representationally correct if p is true. There is a debate in philosophy of language/mind whether representational correctness is normative. Many anti‐normativists have claimed that it is not and correctness is equivalent to truth (for an overview, see Glüer & Wikforss, 2018, section 2.1). In response, Rosen has convincingly argued that this line of thought doesn't properly distinguish between correctness qua a property and the non‐normative correct‐making feature, truth (Rosen, 2001, p. 619). What is important here is that even if correctness is normative, it's not therefore ipso facto deontic or evaluative. In recent metanormative theory, philosophers have started distinguishing between three broad families of normative notions: the deontic, the evaluative, and the fitting. Correctness might be thought to belong in the fittingness family (Berker, 2022). The idea that representational correctness is a variety of the fitting is an unexplored idea and fruitful terrain for further research.

^2^
Wittgenstein later adds that talk of interpretation should be restricted to substituting one *expression* of a rule for another (#201).

^3^
On Wittgenstein's notion of grammar see Glock (1996, pp. 150‐155), Fogelin (2009, Ch. 2), Forster (2004, Ch. 1).

^4^
It's essential to understand that skeptical challenge pertains to the *linguistic* correctness of the use of ‘plus’ and not to the *factual* correctness of the answer. In other words, the question is whether the word was correctly used and not whether the answer was mathematically correct. Right at the beginning of his discussion Kripke writes: I am confident, perhaps after checking my work, that 125 is the correct answer. It is correct both in the *arithmetical* sense that 125 is the sum of 68 and 57, and in the *metalinguistic* sense that ‘plus’, as I intended to use that word in the past, denoted a function which, when applied to the numbers called ‘68’ and ‘57’, yields the value 125. … Now suppose I encounter a bizarre sceptic. This skeptic questions my certainty about my answer, in what I just called the ‘metalinguistic’ sense (Kripke, 1982, 8)
In this passage, linguistic correctness = the “metalinguistic” sense and factual correctness = arithmetical sense. For more on the contrast between them see Ginsborg (2022), Reiland (2021).

^5^
Here it is essential to understand that the guidance and justification pertain to *meaningful* use and not *correct* use. These are not the same thing and to be fully linguistically justified in your use by your own lights doesn't require your use to be correct. This is easiest to see in the case of a meaningful expression that is non‐representational. For example, the meaning of ‘Goodbye!’ in my idiolect is for using when I'm parting from you. This meaning guides me and fully justifies my using ‘Goodbye!’ even when I'm delivering you to your door for a surprise party, fully knowing that I'm not actually parting from you (Kaplan, MS: 16‐17).
